# Independent Role of Underlying Kidney Disease on Renal Prognosis of Patients with Chronic Kidney Disease under Nephrology Care

**DOI:** 10.1371/journal.pone.0127071

**Published:** 2015-05-20

**Authors:** Luca De Nicola, Michele Provenzano, Paolo Chiodini, Silvio Borrelli, Carlo Garofalo, Mario Pacilio, Maria Elena Liberti, Adelia Sagliocca, Giuseppe Conte, Roberto Minutolo

**Affiliations:** 1 Nephrology Division School of Medicine-Second University of Naples, Naples, Italy; 2 Medical Statistics Unit School of Medicine-Second University of Naples, Naples, Italy; University of Perugia, ITALY

## Abstract

Primary kidney disease is suggested to affect renal prognosis of CKD patients; however, whether nephrology care modifies this association is unknown. We studied patients with CKD stage I-IV treated in a renal clinic and with established diagnosis of CKD cause to evaluate whether the risk of renal event (composite of end-stage renal disease and eGFR decline ≥40%) linked to the specific diagnosis is modified by the achievement or maintenance in the first year of nephrology care of therapeutic goals for hypertension (BP ≤130/80 mmHg in patients with proteinuria ≥150 mg/24h and/or diabetes and ≤140/90 in those with proteinuria <150 mg/24h and without diabetes) anemia (hemoglobin, Hb ≥11 g/dL), and proteinuria (≤0.5 g/24h). Survival analysis started after first year of nephrology care. We studied 729 patients (age 64±15 y; males 59.1%; diabetes 34.7%; cardiovascular disease (CVD) 44.9%; hypertensive nephropathy, HTN 53.8%; glomerulonephritis, GN 17.3%; diabetic nephropathy, DN 15.9%; tubule-interstitial nephropathy, TIN 9.5%; polycystic kidney disease, PKD 3.6%). During first year of Nephrology care, therapy was overall intensified in most patients and prevalence of main therapeutic goals generally improved. During subsequent follow up (median 3.3 years, IQR 1.9-5.1), 163 renal events occurred. Cox analysis disclosed a higher risk for PKD (Hazard Ratio 5.46, 95% Confidence Intervals 2.28–10.6) and DN (1.28,2.99–3.05), versus HTN (reference), independently of age, gender, CVD, BMI, eGFR or CKD stage, use of RAS inhibitors and achievement or maintenance in the first year of nephrology care of each of the three main therapeutic goals. No interaction was found on the risk of CKD progression between diagnostic categories and month-12 eGFR (P=0.737), as with control of BP (P=0.374), Hb (P=0.248) or proteinuria (P=0.590). Therefore, in CKD patients under nephrology care, diagnosis of kidney disease should be considered in conjunction with the main risk factors to refine renal risk stratification.

## Introduction

The 2012 update of KDIGO (Kidney Disease: Improving Global Outcomes) guideline recommends considering the cause of kidney disease as modifier of CKD prognosis in addition to albuminuria and estimated glomerular filtration rate (eGFR) [1]. However, NKF-KDOQI (National Kidney Foundation-Kidney Disease Outcomes Quality Initiative) guideline workgroup has more recently highlighted that the independent prognostic role of the cause of CKD still remains undefined and needs more studies prior to be incorporated in the CKD classification [2].

Early studies on renal prognosis have shown that albuminuria level is equally or more predictive than the cause of CKD [3–6]. These studies examined patients in the early 90’s, therefore being poorly informative for today practice. Mean age was in fact around 50 years while now most CKD patients referred to renal clinics are over 65 years [1,7], diabetic nephropathy was poorly or not represented at all while it is now a main cause of CKD [8], and use of agents inhibiting the renin-angiotensin system (RAS), currently considered as the first-choice drugs in CKD, was not mentioned or limited to a minority of patients. A recent post-hoc analysis of the randomized controlled trial (RCT) SHARP (Study of Heart and Renal Protection), originally aimed at evaluating the effect of ezetimibe-simvastatin in CKD, has provided more insights into this topic [9]. Authors reported that patients with cystic kidney disease had higher risk of end stage renal disease (ESRD) as compared with other diagnosis groups. The study, however, hardly allows to estimate the renal risk associated with each specific diagnosis in the “real world” of tertiary nephrology care because investigators excluded patients with coronary artery disease that account for a substantial quote of contemporary patient population in renal clinics [1,7], and no information was provided on length and efficacy of nephrology intervention prior to the start of survival analysis. Analysis of the contribution of the single specific diagnosis to the progression of CKD was also limited because the largest study group “other diagnoses” (56% of whole population) was constituted by pooling together heterogeneous diagnostic categories, such as hypertensive disease and pyelonephritis, with undefined or unknown diagnoses (as many as 35% and 23% of the group, respectively).

From our outpatient clinic dedicated to CKD-ND, we selected patients with diagnosed primary renal disease to evaluate whether renal prognosis linked to the specific cause of CKD changes according to the degree of control of hypertension, proteinuria and anemia, that have been identified as the main modifiable determinants of renal events [1,7,10–14].

## Methods

This is a observational cohort study based on a prospective database including all the adult patients, no dialysis/no kidney transplant, referred to our outpatient clinic dedicated to the conservative treatment of CKD. To this clinic are referred patients with non-dialysis CKD and no acute disease, such as active glomerulonephritis or acute interstitial nephritis. Each patient was seen by the same nephrologist at all visits. The study was approved by the Institutional Review Board (Second University of Naples) and patients gave written consent to use their clinical data.

For the purposes of the study, we considered eligible all consecutive patients referred from 01/2000 to 12/2010 with CKD stage I to IV documented from at least 3 months. We excluded patients with undefined cause of CKD, patients not completing the first year of nephrology care (lost to follow up), those with active malignancy, evidence of acute kidney injury in the 3 months prior to the first visit, and patients with poor compliance to therapy (missing rate of pills in the two weeks prior to any visit ≥20%). Selected patients were classified in five diagnostic categories according to the diagnosis of underlying renal disease (see S1 Text): hypertensive nephropathy (HTN), diabetic nephropathy (DN), glomerulonephritis (GN), tubule-interstitial nephropathy (TIN), and polycystic kidney disease (PKD).

At each visit, nephrologists performed the physical examination, including the measurement body weight and BP, reviewed lab results and prescribed therapy including personalized diet with limited amount of protein (0.3–0.8 g protein/kg/day) and salt (<6 g NaCl/day) where appropriate. Laboratory protocols were standardized with in-house analysis of samples. Twenty-four hour urine collection was obtained to quantify proteinuria and to evaluate adherence to the prescribed restriction of protein and salt intake. Collection was considered inaccurate, and repeated, if the creatinine excretion was outside of the 60 to 140% range of the value calculated according to Dwyer and Kenler [15]. GFR was estimated (eGFR) by the four-variable MDRD equation. BP was measured in a sitting position 3 times at 5-min intervals. Variables collected at the referral nephrology visit (baseline) and after one year (12-month visit) were used for analyses.

Main modifiable risk factors of CKD progression were defined at goal according to the following thresholds: blood pressure lower or equal to 130/80 mmHg in CKD patients with abnormal proteinuria (≥150 mg/24h) and/or diabetes and lower or equal to 140/90 mmHg in patients without proteinuria and/or diabetes, hemoglobin (Hb) higher or equal to 11 g/dL, proteinuria lower or equal to 0.5 g/24 h.

### Statistical analysis

Continuous variables were reported as either mean and standard deviation (SD) or median and interquartile ranges (IQR) according to their distribution. Intra-group differences from baseline to month-12 visit were analyzed using paired Student’s t-test or Wilcoxon test, whereas inter-group differences were tested by either one-way ANOVA or Kruskal-Wallis test. Categorical variables were reported as percentages and analyzed by means McNemar test (Intra-group differences) or chi-squared test (inter-group differences).

Follow up for the renal survival analysis lasted from month 12 of nephrology to December 31^st^ 2013. To evaluate the change of renal function over time, we calculated the slope of eGFR (ml/min/year) by means of the ordinal least square method and using all the eGFR values after month-12 visit. Endpoint of the study (renal endpoint) was the composite of ESRD, that is, start of chronic dialysis treatment or kidney transplant, or incidence of eGFR decline ≥40%, that is now accepted as adequate surrogate of hard renal endpoint [16–18]. Unadjusted association between diagnoses and renal endpoint was assessed by means of competing risk approach and Gray test because death and renal event are competitive events [19,20]. The same approach was used to test the unadjusted association between diagnoses and all-cause death before ESRD. A multivariable Cox proportional hazards model was used to estimate hazard ratios (HRs) and 95% confidence intervals (CIs) of the primary composite renal endpoint in the five diagnostic categories (using HTN as reference). We calculate HRs using Cox models because the cause-specific relative hazards are more applicable for studying the cause of diseases in the case of a competing event [21,22]. Similar analysis was used to estimate HRs for the control of each of the main modifiable risk factors (BP, Hb, proteinuria): (1) *Unachieved at month 12* (reference), that includes all patients that were not at goal by month 12, (2) *Achieved only at month 12*, that includes patients that improved their status being not at goal at referral, (3) *Achieved at both visits*, that includes patients at goal at referral and month-12 visit. The separate analysis of the latter two categories allows to discriminate whether achievement of goal at month 12 influences renal risk differently if patients reached the goal at month 12 (improvers) or they maintained it at both visits (stable at goal). To test the independent prognostic role of diagnosis and goal status, the two models were also tested combined. Models were either unadjusted or adjusted by including a priori the value at month 12, that is, the starting point of survival analysis, of main variables that potentially modify renal prognosis (age, gender, history of CV disease, BMI, eGFR, use of RAS inhibitors). Sensitivity analyses were also performed by considering different threshold values for goal definition. We further investigated whether the goal status modulated the prognostic role of renal diagnosis on CKD progression by analyzing their interaction; to test it, we added their interaction to the fully adjusted model. Because of the major role of renal function as main determinant of renal survival, in the Cox models, we not only included month-12 eGFR as covariate but also tested the interaction of eGFR with diagnostic categories in the prediction of renal outcome. Finally, we evaluated whether HRs changed after stratifying the Cox model by CKD stage and removing eGFR as covariate (sensitivity analysis).

In the Cox model, the contribution of each covariate to the model fit was estimated as percentage reduction of R^2^ value of the model resulting from omitting each variable in turn from the full model [23]. We calculated R^2^s according to Nagelkerke [24]. Proportional hazards assumptions were evaluated using Schoenfeld residual tests for overall model and for each cause of renal disease [25]. A two-tailed p value <0.05 was considered significant. Data were analyzed using SAS version 9.2 (SAS Inc., Cary, NC).

## Results

**Fig 1:**
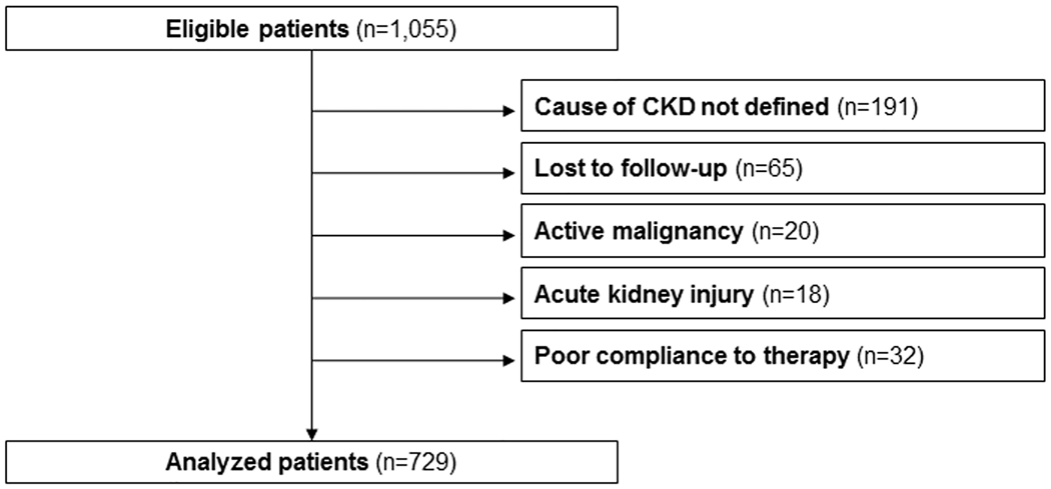
Flow chart of study cohort.

We studied 729 out of 1,055 eligible patients; 65 patients did not complete the first year because of the change of renal clinic (n = 63) or start of dialysis (n = 2), while no patient died (Fig 1). Most frequent cause of CKD was HTN (53.8%,95%CI 50.2–57.4) followed by GN, DN and TIN (17.3%,95%CI 14.5–20.0; 15.9%,95%CI 13.3–18.6; 9.5%, 95%CI 7.3–11.6, respectively), while PKD accounted for 3.6%,95%CI 2.2–4.9 of patients. DN patients accounted for about half of diabetics (116/253), the vast majority having type 2 diabetes.

### First year of nephrology care

As reported in Table 1, mean age substantially differed with higher prevalence of subjects over 65 years in HTN and DN (77.3% and 49.1%) versus TIN, GN and PKD (31.9%, 20.6%, 15.4%). Obesity (BMI >30 kg/m^2^) was more prevalent in DN (39.1%) and HTN (37.2%) than in GN, TIN and PKD (29.4, 22.9 and 23.1%, respectively). DN and HTN patients were also characterized by higher prevalence of CV disease mainly represented by coronary artery disease.

**Table 1:** Clinical characteristic of cohort, overall and stratified for cause of CKD, at referral visit.

	Overall (n = 729)	HTN (n = 392)	DN (n = 116)	GN (n = 126)	TIN (n = 69)	PKD (n = 26)	*P*
**Age (years)**	63.6±15.1	70.8±8.8	65.0 ±10.2	48.3±16.4	55.1 ±17.1	46.0 ±16.0	<0.001
**Male gender (%)**	59.1	60.2	62.1	65.1	44.9	38.5	0.012
**Body Mass Index (kg/m** ^**2**^ **)**	28.9±5.3	29.5±5.3	29.6±6.1	27.9±4.1	27.2±5.2	27.0±3.9	<0.001
**Smoker (%)**	20.6	14.3	25.0	31.0	24.6	34.6	<0.001
**Diabetes mellitus (%)**	34.7	28.3	100	15.1	10.1	0	<0.001
**CV disease (%)**	44.9	54.8	60.3	22.2	13.0	19.2	<0.001
**LVH (%)**	58.9	70.1	70.7	34.1	34.1	30.8	<0.001
**CKD stage (%)**							<0.001
**Stage I**	5.8	1.3	3.4	19.8	7.2	11.5	
**Stage II**	15.0	9.4	12.1	32.5	17.4	19.2	
**Stage IIIa**	29.1	35.2	31.0	13.5	23.2	19.2	
**Stage IIIb**	32.5	37.5	31.9	19.0	31.9	26.9	
**Stage IV**	17.7	16.6	21.6	15.1	20.3	23.1	

Data are mean±SD or % patients. HTN, hypertensive nephropathy; DN, diabetic nephropathy, GN, glomerulonephritis; TIN, tubulointerstitial nephropathy; PKD, autosomal polycystic kidney disease; CV, cardiovascular; LVH, left ventricular hyperthrophy

Table 2 describes main laboratory parameters and BP levels. At referral, HTN and DN were characterized by lower eGFR and Hb levels and higher BP while the GN group showed higher eGFR. As expected, proteinuria was higher and serum albumin lower in GN. During the first 12 months of nephrology care, eGFR did not change (from 48.1±20.7 to 47.4±21.1 mL/min/1.73m^2^, P = 0.11) overall, with only DN patients showing a slight but significant reduction. Similarly, proteinuria decreased overall with more marked reduction in GN.

**Table 2:** Clinical and laboratory parameters at baseline and at month-12 visit in patients stratified for cause of CKD.

	HTN		DN		GN		TIN		PKD	
	*Baseline*	*Month 12*	*Baseline*	*Month 12*	*Baseline*	*Month 12*	*Baseline*	*Month 12*	*Baseline*	*Month 12*
**eGFR (mL/min/1.73m** ^**2**^ **)** ^**a**^ ^,^ ^**b**^	44.4±14.9	44.2 ±14.9	45.2±19.1	42.0±19.9*	61.6±28.4	59.8±28.9	48.6±22.9	51.1±24.8	50.3±25.7	49.1±28.2
**Uric acid (mg/dL)** ^**a**^	6.5±1.8	6.3±1.7*	6.4±1.7	6.4±1.9	6.4±1.8	6.3±1.6	5.9±1.8	5.8±1.8	5.1±1.6	5.5±2.0
**Phosphate (mg/dL)** ^**b**^	3.67±0.74	3.62±0.68	3.84±0.85	3.94±0.81	3.89±0.82	3.80±0.59	3.87±0.66	3.88±0.78	4.23±0.77	4.10±0.88
**PTH (pg/mL)** ^**a**^	94 ±67	90±68	65±41	74±52	73±54	94±77*	85±52	95±94	57±39	87±57
**Albumin (g/dL)** ^**a**^ ^,^ ^**b**^	4.06±0.47	4.10±0.44	3.84±0.61	3.97±0.41*	3.72±0.80	3.95±0.53*	4.02±0.56	4.15±0.5	4.18±0.47	4.13±0.40
**Cholesterol (mg/dL)** ^**a**^ ^,^ ^**b**^	187±40	179±37*	190±51	174±35*	214±72	185±40*	202±39	194±43	195±41	185±35
**TG (mg/dL)** ^**a**^ ^,^ ^**b**^	134±68	131±70	153±80	148±75	162±99	145±86*	147±107	128±59	108±40	107±52
**Hemoglobin (g/dL)** ^**a**^ ^,^ ^**b**^	12.8±1.8	13.1±1.6*	12.5±2.0	12.5±1.6	13.5±2.1	13.1±1.8*	13.3±2.1	13.6±1.7*	13.1±1.3	12.8 ±1.2
**Proteinuria (mg/24h)** ^**a**^ ^,^ ^**b**^	120 (50–250)	120 (30–300)	358 (148–1675)	290 (100–1370)	1.550 (500–4000)	600* (200–1500)	172 (60–500)	190 (30–400)	179 (40–300)	125 (10–370)
**UNaV (mEq/24h)**	145±66	147±61	152±61	151±72	152±70	164±64	148±72	147±69	142±54	146±45
**UUN (g/24h)**	9.0±4.1	9.1±3.7	9.7±4.9	9.0±4.0	10.0±3.9	10.3±4.0	9.6±3.7	8.1±3.1	11.1±3.0	8.9±3.4
**Systolic BP (mmHg)** ^**a**^ ^,^ ^**b**^	144±22	137±19*	149±26	142 ±19*	136±20	128±17*	129±19	128±20	138±21	126 ±15*
**Diastolic BP (mmHg)** ^**a**^ ^,^ ^**b**^	80±12	77±12*	78±12	75±13*	83±11	78±10*	81±11	80±11	89±10	82±8*

Data are mean±SD or median and IQR. HTN, hypertensive nephropathy; DN, diabetic nephropathy, GN, glomerulonephritis; TIN, tubulointerstitial nephropathy; PKD, autosomal polycystic kidney disease. PTH, parathyroid hormone; TG, triglycerides; UnaV, urinary sodium excretion; UUN, urinary excretion of urea nitrogen; BP, blood pressure.

^a^ P for trend <0.05 for basal values;

^b^ P for trend <0.05 for month-12 values

Overall, systolic and diastolic BP decreased by 6 mmHg (IQR -20 to +8) and 2 mmHg (IQR -10 to +5), respectively. Major changes of BP levels were observed in all groups but TIN which was characterized by lower BP at baseline. Also lipid profile improved from baseline to month-12 visit with changes being consistent in all subgroups though with different extent. Hb levels increased in HTN and TIN patients while slightly decreased in GN. According to the observed changes, prevalence of main therapeutic goals generally improved with the exception of proteinuria goal whose frequency significantly increased only in GN (Table 3). This was associated with intensification of overall therapy in most patients (Table 4).

**Table 3:** Control of main modifiable factors at baseline and month-12 visit.

	Overall (n = 729)	HTN (n = 392)	DN (n = 116)	GN (n = 126)	TIN (n = 69)	PKD (n = 26)	P
**BP goal**							
*** Baseline***	37.2 (33.7–40.7)	36.0 (31.2–40.7)	28.4 (20.2–36.7)	39.7 (31.1–48.2)	55.1 (43.3–66.8)	34.6 (16.3–52.9)	0.009
*** Month 12***	50.3* (46.7–54.0)	49.7* (44.8–54.7)	32.8 (24.2–41.3)	58.7* (50.1–67.3)	59.4 (47.8–71.0)	73.1* (56.0–90.1)	<0.0001
**Hb goal**							
*** Baseline***	84.5 (81.9–87.1)	84.4 (80.8–88.0)	75.7 (67.9–83.5)	91.3 (89.4–96.2)	81.4 (72.2–90.6)	100	0.002
*** Month 12***	89.7* (87.5–91.9)	91.1* (88.3–93.9)	87.0* (80.9–93.1)	86.5 (80.5–92.5)	91.4* (84.8–98.0)	92.3 (82.1–100)	0.471
**Uprot goal**							
*** Baseline***	70.7 (67.4–74.0)	86.0 (82.5–89.4)	57.4 (48.4–66.4)	27.0 (19.2–34.7)	75.4 (65.2–85.5)	100	<0.0001
*** Month 12***	72.2 (69.0–75.5)	83.2 (79.5–86.9)	59.1 (50.1–68.1)	42.9* (34.2–51.5)	81.2 (71.9–90.4)	84.0 (69.6–98.4)	<0.0001

Data are % of patients and (95%CI). BP, blood pressure (mmHg); Hb, hemoglobin (g/dL); Uprot, proteinuria (g/24h).

* P<0.05 versus baseline.

See Methods for definition of goal values

**Table 4:** Therapy at baseline and month-12 visit.

	Overall (n = 729)	HTN (n = 392)	DN (n = 116)	GN (n = 126)	TIN (n = 69)	PKD (n = 26)	P
**LSD (%)**							
*** Baseline***	22.8	25.6	18.0	19.3	23.1	16.7	0.354
*** Month 12***	26.2	26.4	31.5*	19.3	29.2	25.0	0.301
**BP lowering drugs (n)**							
*** Baseline***	2.2±1.3	2.5±1.2	2.5±1.2	1.7 ±1.3	1.0±1.1	1.6±1.6	<0.0001
*** Month 12***	2.7±1.4*	2.8±1.2*	3.3±1.5*	2.5±1.4*	1.5±1.4*	2.0±1.4*	<0.0001
**RAS inhibitors (%)**							
*** Baseline***	74.9	82.1	77.4	67.5	54.3	69.2	<0.0001
*** Month 12***	85.3*	88.8*	75.6	88.9*	60.0*	76.9	<0.0001
**B-blockers (%)**							
*** Baseline***	27.4	33.9	30.4	16.7	5.8	24.0	<0.0001
*** Month 12***	36.7*	41.1*	46.1*	27.8*	14.5*	32.0	<0.0001
**CCBs (%)**							
*** Baseline***	34.9	39.0	48.7	20.6	17.4	28.0	<0.0001
*** Month 12***	43.9*	48.7*	60.9*	26.2	24.6	32.0	<0.0001
**Furosemide (%)**							
*** Baseline***	23.1	25.0	33.9	19.0	8.7	4.0	<0.0001
*** Month 12***	34.3*	34.4*	51.3*	34.9*	11.6	12.0	<0.0001
**ESA (%)**							
*** Baseline***	3.0	2.6	0.9	5.6	4.3	3.8	0.256
*** Month 12***	14.4*	14.3*	19.1*	12.7*	12.9*	7.7	0.482
**Iron supplement (%)**							
*** Baseline***	4.1	4.3	3.5	1.6	8.7	4.0	0.210
*** Month 12***	18.2*	16.3*	26.1*	17.5*	14.5	24.0	0.136
**Statins (%)**							
*** Baseline***	30.0	31.1	45.2	26.2	10.1	16.9	<0.0001
*** Month 12***	43.7*	45.7*	55.7*	38.1*	29.0*	28.0	0.002
**Vitamin D (%)**							
*** Baseline***	4.0	3.1	0.9	7.1	8.7	4.0	0.026
*** Month 12***	13.9*	12.5*	9.6*	21.4*	14.5	16.0	0.074
**P binders (%)**							
*** Baseline***	0.3	0.3	0	0.8	0	0	0.769
*** Month 12***	2.1*	1.3	4.3	0.8	4.3	4.0	0.115

Data are mean±SD or % of patients and (95%CI). RAS, renin-angiotensin system; BP, blood pressure; LSD, low salt diet (UNaV <100 mmol/24h); ESA, erythropoiesis stimulating agents.

* P<0.05 versus baseline

### Renal survival

**Fig 2:**
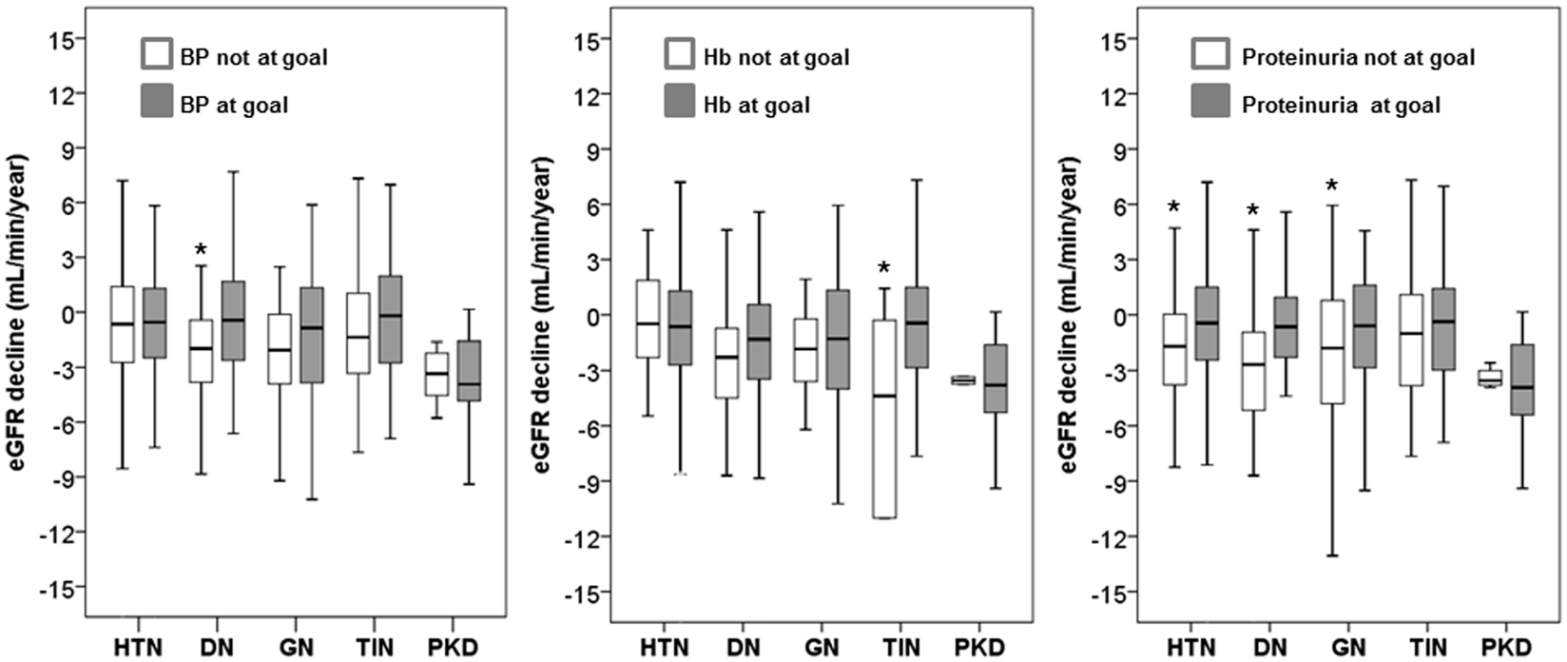
Box plots of decline of renal function, estimated as eGFR slope, in the five categories of diagnosis and by goal status at the beginning of follow up for renal survival (month 12 visit). Limits of boxes are 25th and 75th percentiles, with the thick line across the boxes being the median value. Whiskers indicate either the minimum and maximum values or a distance of 1.5 times the interquartile range from the edge of the box (whichever distance is smaller). *P<0.05 versus achieved goal. See Methods for definition of goal values.

Follow up for CKD progression started after month 12 and lasted a median of 3.3 years (IQR 1.9–5.1). We calculated the slope of eGFR during this period by using a median of 6 (range 3–9) serum creatinine values. In the whole population, eGFR decline was -0.99 mL/min/year, IQR from -3.33 to +1.14. As compared to HTN (-0.62, IQR from -2.70 to +1.30 mL/min/year), a greater decrease was observed in DN (-1.39, IQR from -3.73 to +0.43 mL/min/year, P = 0.016) and PKD (-3.70, IQR from -5.16 to -1.61 mL/min/year, P<0.001), while no significant difference was observed in GN (-1.34, IQR from -3.85 to +1.33 mL/min/year, P = 0.074) and TIN (-0.77, IQR from -3.09 to +1.42 mL/min/year, P = 0.983). Fig 2 shows the unadjusted association between goal status at month 12 and subsequent eGFR decline in each diagnostic category; all patients but those with TIN and PKD had a slower CKD progression when month-12 proteinuria was at goal. Less consistent association was found for the other two factors.

**Fig 3:**
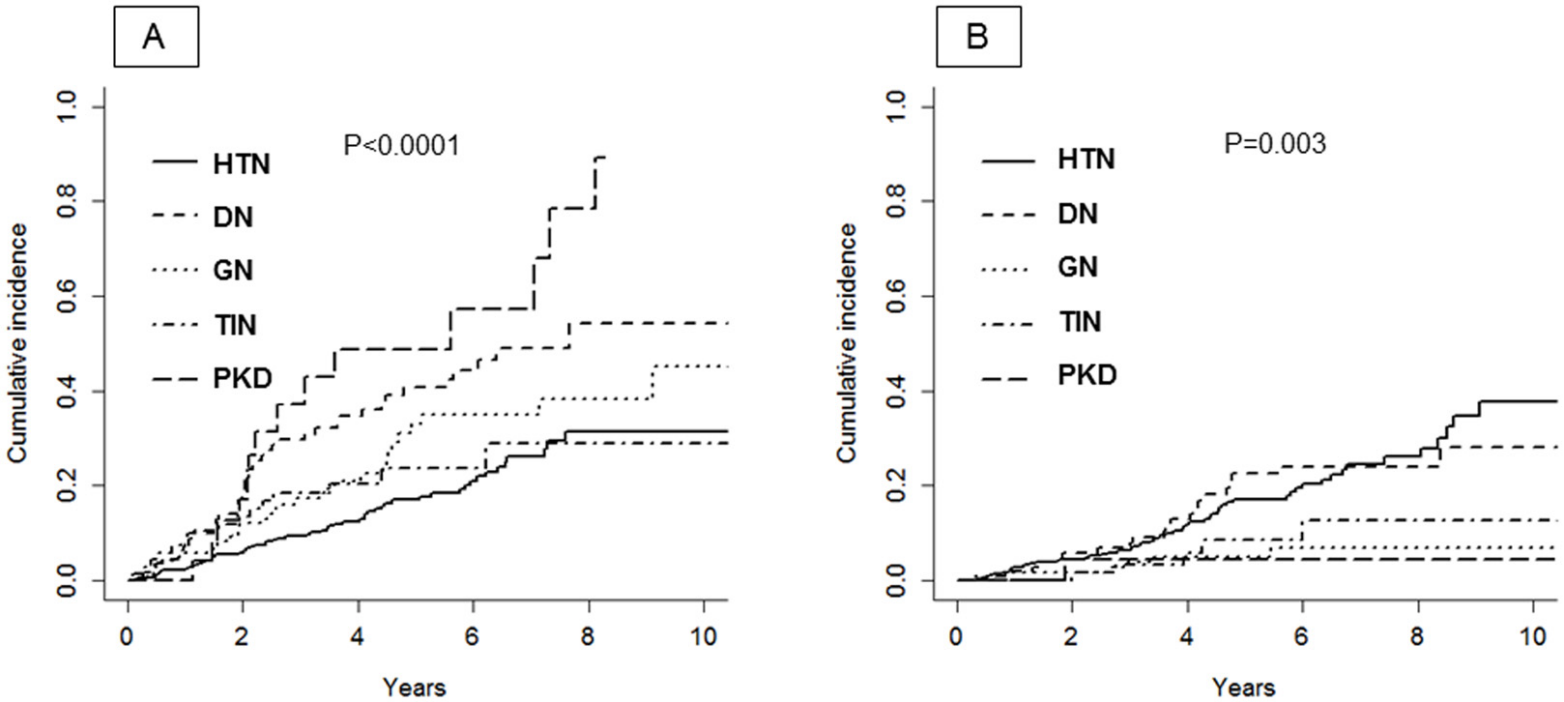
Cumulative incidence after month-12 visit of renal event (panel A) and all-cause death before ESRD (panel B).

We registered 163 renal events (dialysis, n = 60; eGFR decline ≥40%, n = 103) and 90 all-cause deaths prior to ESRD, 70% being of CV nature. The incidence rate of the composite renal endpoint increased from stage I-II to stage IV (from 3.7 to 15.4/100 pts/y, P<0.001); overall rate was 6.1/100 pts/y (95%CI 5.2–7.1), that is, almost the double than that of death (3.1/100 pts/y, 95%CI 2.5–3.8). Incidence of renal endpoint was higher in DN and PKD, while higher mortality before ESRD occurred in DN and HTN (Fig 3).

Table 5 shows cause-specific hazards of the primary combined renal event. Renal risk was consistently higher for DN and PKD in unadjusted as adjusted models. In adjusted models, the protective effect of achieved therapeutic goal did not differ across the three different parameters examined and was analogous when considering the two visits or the latter visit only. Similar results were obtained when different threshold values for goal definition were considered (see S2 Table). No interaction on the risk of CKD progression was found between diagnostic categories and month-12 eGFR (P = 0.737), as with control of BP (P = 0.374), Hb (P = 0.248) or proteinuria (P = 0.590). Furthermore, HRs of diagnostic categories did not change when the full model was stratified by CKD stage in the sensitivity analysis, (DN 2.16, GN 1.39, TIN 1.97, PKD 5.12). According to R^2^ reduction analysis, testing the individual contribution to the variance of the fully adjusted model, the prognostic role of diagnosis and proteinuria goal resulted similarly predominant versus control of BP or Hb (Table 5). In all Cox models, the proportional hazards assumption was satisfied, as shown by Schoenfeld residual. In particular, in model 2, P value of proportional hazards assumption was 0.334 for the global test and 0.09 for diagnostic categories.

**Table 5:** Multivariable Cox models of determinants of the combined renal endpoint.

	Unadjusted		Model 1		Model 2		
	HR	95% CI	HR	95% CI	HR	95% CI	R^2^R (%)
**Cause of kidney disease**							15.0
**HTN**	Ref.		Ref.		Ref.		
**DN**	2.72	1.84–4.03	2.44	1.63–3.66	1.96	1.28–2.99	
**GN**	1.54	1.00–2.36	2.38	1.46–3.87	1.39	0.83–2.31	
**TIN**	1.33	0.75–2.34	1.61	0.86–3.01	1.80	0.95–3.41	
**PKD**	3.64	2.03–6.53	5.01	2.57–9.74	5.46	2.28–10.6	
**Blood pressure goal**							8.0
***Unachieved at month 12 (n = 361)***	Ref.		Ref.		Ref.		
***Achieved only at month 12 (n = 184)***	0.57	0.39–0.84	0.52	0.35–0.78	0.59	0.39–0.89	
***Achieved at both visits (n = 182)***	0.35	0.22–0.56	0.42	0.26–0.68	0.46	0.28–0.76	
**Hemoglobin goal**							7.0
***Unachieved at month 12 (n = 74)***	Ref.		Ref.		Ref.		
***Achieved only at month 12 (n = 74)***	0.55	0.32–0.95	0.45	0.26–0.79	0.48	0.27–0.85	
***Achieved at both visits (n = 579)***	0.34	0.23–0.50	0.42	0.28–0.64	0.44	0.29–0.68	
**Proteinuria goal**							13.0
***Unachieved at month 12 (n = 202)***	Ref.		Ref.		Ref.		
***Achieved only at month 12 (n = 73)***	0.35	0.19–0.65	0.38	0.20–0.72	0.44	0.23–0.83	
***Achieved at both visits (n = 452)***	0.33	0.24–0.46	0.38	0.27–0.54	0.43	0.29–0.62	

Model 1: diagnoses and goals are separately adjusted for main covariates (age, gender, history of CV disease, BMI, eGFR, use of RAS inhibitors). Model 2: fully adjusted (adjusted for main covariates of model 1 plus diagnoses and therapeutic goals). See Methods for definition of goal values. HTN, hypertensive nephropathy; DN, diabetic nephropathy; GN, glomerulonephritis; PKD, autosomal polycystic kidney disease; TIN, tubulointerstitial nephropathy. R^2^R, R^2^ reduction.

## Discussion

Optimizing stratification of renal risk in the population of CKD patients referred to tertiary nephrology care is a central issue of nephrology research. Indeed, the large and growing population of CKD-ND patients is, and will be, hardly managed by the limited, and shrinking, human and economic resources dedicated to this specialty. Identification of high-risk patients should therefore allow nephrologists to better focus on patients that require close monitoring and treatment as well as timely planning of renal substitutive therapy. The current study adds to the knowledge on the prognostic role of the cause of CKD by providing original information in CKD patients under nephrology care with defined diagnosis of underlying disease and prolonged follow up.

We found that diagnosis of DN and PKD was associated with faster decline of eGFR and higher risk of the composite renal event persisting in adjusted analyses. The absence of any interaction between the distinct diagnostic categories and optimal control of BP, proteinuria or Hb indicates the independence of renal risk inherent to the cause of CKD. Noteworthy, the prognostic role of primary diagnosis was similarly unmodified by eGFR or CKD stage, thus indicating that underlying renal disease should be considered in the stratification of renal risk regardless renal function level.

On the other hand, the significant prognostic role of the achievement or maintenance of goal for the three examined modifiable risk factors in the multi-adjusted Cox analysis, as well as the lack of any interaction with diagnosis, may suggest that better control allows attenuation of the risk in each diagnostic category as compared to HTN.

A period as long as 15 to more than 20 years elapsed between the few early studies on this issue, that were therefore poorly representative of contemporary CKD population [3–6], and the recent report on SHARP population [9]. This secondary SHARP analysis evidenced that, as compared with the other diagnostic categories, PKD patients had a 3-fold higher risk of ESRD and an unexplained lower risk of mortality while patients with diabetic nephropathy had ESRD rates similar to those observed in the other non-PKD participants and were much more likely to die before ESRD. In this study, we estimated cause-specific hazards to assess the effect of diagnosis on renal risk because this analysis is adequate in the case of the competing event of death [22,23]. Conversely, we did not specifically address the relevance of the cause of CKD to the risk of death because a proper evaluation of this outcome, that is, by multi-adjusted analysis as made for renal endpoint, would have required a higher number of events. In this regard, several studies have reported that in patients followed by nephrologist the risk of death is relatively low while mortality largely overcomes ESRD in unreferred patients [7,14,26–29]. This finding may be related to the larger prevalence in renal clinics of “true” chronic renal disease whose “natural” fate is ESRD and/or to the nephrology intervention. The latter hypothesis cannot be proven by our observational study; however, it is supported by a study of a cohort of elderly diabetic CKD patients where mortality rates were lower when patients were treated by nephrologists rather than other specialists [30], as well as by two systematic reviews that have disclosed higher mortality rates in CKD patients referred late to nephrologist [31,32].

Study cohort was characterized not only by a relatively low mortality but also by slow rates of eGFR decline, approximately 1.0 mL/min/year overall, which is not unusual in CKD patients treated in the nephrology setting [7,14,26,33]. Furthermore, a positive association between mortality and progression of renal disease has been repeatedly reported in different cohorts of subjects with and without CKD [18,33–36].

The observation that achievement or maintenance of main therapeutic goals attenuated renal risk is a main and original finding of the study. Noteworthy, the subgroup of patients achieving therapeutic goals at month 12 displayed a risk similar to that of patients spontaneously at target (low risk subjects); these data, therefore, support the positive role of nephrology intervention. The magnitude of the protective effect of target achievement is not unexpected when considering that all enrolled patients were incident, that is, newly referred and treated in a renal clinic dedicated to the conservative therapy of CKD.

More insights into the relationship between nephrology care and renal outcome were provided by the analysis of eGFR slopes as continuous variable for the three therapeutic goals examined (Fig 2). Optimal control of either BP or proteinuria was associated with slower rates of renal function decline in DN patients, thus extending to the setting of daily practice in outpatient renal clinic the results of main RCTs testing the nephroprotective effect of anti-RAS agents on the outcome of type 2 diabetic patients with CKD [37,38]. Conversely, in PKD patients, the faster rate of eGFR decline remained unmodified. Noteworthy, progression rates of the whole PKD group were identical to those detected in SHARP study (-3.7 and -3.8 mL/min/year, respectively); this finding suggests that the relentless progression of PKD is independent of study setting and patient features. Overall, the results of our analyses support the need of more efficacious therapeutic strategies in DN and PKD patients followed in nephrology clinics [8,39–41].

Among the three modifiable factors examined, proteinuria emerged as a main predictor of renal outcome. This was evident in the unadjusted analysis of eGFR slope where the high prognostic value of control of this parameter was consistent in HTN, GN and DN. Similarly, when estimating the hierarchy of prognostic factors in the fully adjusted model for assessment of renal hazards, the prognostic weight of proteinuria resulted to overlap that of primary renal diagnosis. These data, while supporting the new classification of CKD that incorporates proteinuria or albuminuria level in disease staging [1], extend to the population of CKD patients seen in the setting of daily nephrology practice the results of a recent meta-analysis of randomized clinical trials showing that early antiproteinuric response to therapy predicts improved long-term renal survival [42].

Interestingly, similar results on renal prognosis were obtained when considering different therapeutic goal values (see S2 Table). This finding suggests the greater importance of nephrology care *per se* versus specific goals for CKD progression; however, it does not exclude that the level of control may affect CV outcome. This dilemma, discussed in recent studies, commentaries and also addressed by current guidelines [1,43–47], certainly warrants *ad hoc* RCTs to be solved. This holds particularly true for BP control that should be stricter (<130/80 rather than <140/90 mmHg) only in the presence of significant albuminuria or proteinuria and diabetes [1,43,44]; this suggestion is supported by the excess risk of stroke in diabetic patients with BP values < 140/90 vs 130/80 mmHg, independent of the presence of proteinuria [45].

Our study has limitations. First, the cohort was formed only by Caucasian patients. Second, the single-center dimension of this study limits generalizability of results; however, at the same time, it allows a proper analysis of the study objectives by ensuring homogeneity of diagnosis, therapeutic goals and type of intervention. Third, treatment was goal-oriented but many patients did not reach/maintain them; this discrepancy, inherent to the observational studies in clinical practice, does not allow to exactly quantify the change of hazards related to achievement of therapeutic goals. These limitations, and the small sample size as well, indicate the preliminary nature of these results.

In conclusion, in CKD patients followed in the setting of outpatient renal clinic, diagnosis of diabetic nephropathy or polycystic kidney disease is associated with worse renal prognosis independently from the main modifiable determinants of CKD progression, such as blood pressure, proteinuria and hemoglobin. These data suggest that diagnosis of primary renal disease should be considered in conjunction with the main determinants of CKD progression to better refine renal risk stratification in tertiary nephrology care.

## Supporting Information

S1 File Modality of diagnosis of underlying kidney disease. (DOCX)

S1 Table Determinants of renal endpoint by different cut-off values for control of hypertension, anemia and proteinuria. (DOCX)

S1 Dataset (SPSS format). (ZIP)
