# Supplementary material for: Independent Role of Underlying Kidney Disease on Renal Prognosis of Patients with Chronic Kidney Disease under Nephrology Care
Source: PLoS One. 2015 May 20;10(5):e0127071. doi: 10.1371/journal.pone.0127071 (PMC4439030; doi:10.1371/journal.pone.0127071)
Supplement: S1 File — (DOCX) [file pone.0127071.s001.docx]

**DIAGNOSIS OF UNDERLYING KIDNEY DISEASE**

Diagnosis of primary kidney disease was made by participating nephrologists at the beginning of nephrology care. For the study, all diagnoses were revised by the three veteran nephrologists of the group (GC, LDN, RM) that analyzed the clinical charts in anonymous form and independently adjudicated diagnosis for each patient. Agreement of diagnosis among the three scorers was assessed by Kappa statistics with a minimum value of 0.75 required to finalize the diagnosis. If Kappa coefficient resulted <0.75, cause was defined as unknown. Patients not considered eligible for the study because of unknown diagnosis or lack of agreement were 152.

Diagnostic criteria of underlying nephropathy were the following:

**Diabetic**: Duration of diabetes ≥ 12 years and albuminuria or proteinuria, and/or diabetic retinopathy; absence of hematuria [1-5]

**Hypertensive:** History of at least 8 years of high blood pressure before the onset of any renal impairment and exclusion of other renal diseases [6-11]

**Glomerulonephritis:** Kidney biopsy; clinical or laboratoristic history of glomerulonephritis: macroscopic hematuria that occurs 1-2 days after an infection; nephritic syndrome that occurs a few weeks after an infection; nephrotic syndrome; medical history and clinical signs suggestive of secondary glomerulonephritis [12-13]

**Policystic Kidney Disease:** Clinical signs; positive familiar history; diagnostic imaging [14-15]

**Tubulo-Interstitial:** CKD due to post-renal causes (nephrolithiasis, infections, pyelonephritis); clinical signs (salt-wasting nephropathy); diagnostic imaging (scarring, hydronephrosis); drugs; systemic disease (sarcoidosis, Sjögren’s syndrome, urate nephropathy)

**Unknown:** None of previous features or lack of agreement among the three veteran nephrologist

REFERENCES

1. Sharma SH, Bomback AS, Radhakrishnan J, Herlitz LC, Stokes MB, Markowitz GS, and D’Agati VD. (2013) The Modern Spectrum of Renal Biopsy Findings in patients with Diabetes. Clin J Am Soc Nephrol 8: 1718-1724.
2. Liang S, Zhang XG, Cai GY, Zhu HY, Zhou JH, Wu J, Chen P, Lin S, Qiu Q, Chen XM. (2013) Identifying Parameters to Distinguish Non-Diabetic Renal Diseases from Diabetic Nephropathy in Patients with Type 2 Diabetes Mellitus: A Meta-Analysis*.* PLOS ONE 8: e64184.
3. Mak SK, Gwi1 E, Chan KW, Wong PN, Lo KY, Lee KF and Wong AKM. (1997) Clinical predictors of non-diabetic renal disease in patients with non-insulin dependent diabetes mellitus. Nephrol Dial Transplant 12: 2588-2591.
4. [Zhou J](http://www.ncbi.nlm.nih.gov/pubmed?term=Zhou%20J%5BAuthor%5D&cauthor=true&cauthor_uid=18156459)1, [Chen X](http://www.ncbi.nlm.nih.gov/pubmed?term=Chen%20X%5BAuthor%5D&cauthor=true&cauthor_uid=18156459), [Xie Y](http://www.ncbi.nlm.nih.gov/pubmed?term=Xie%20Y%5BAuthor%5D&cauthor=true&cauthor_uid=18156459), [Li J](http://www.ncbi.nlm.nih.gov/pubmed?term=Li%20J%5BAuthor%5D&cauthor=true&cauthor_uid=18156459), [Yamanaka N](http://www.ncbi.nlm.nih.gov/pubmed?term=Yamanaka%20N%5BAuthor%5D&cauthor=true&cauthor_uid=18156459), [Tong X](http://www.ncbi.nlm.nih.gov/pubmed?term=Tong%20X%5BAuthor%5D&cauthor=true&cauthor_uid=18156459)A. (2008) Differential diagnostic model of diabetic nephropathy and non-diabetic renal diseases. Nephrol Dial Transplant 23: 1940-5.
5. [Chang TI](http://www.ncbi.nlm.nih.gov/pubmed?term=Chang%20TI%5BAuthor%5D&cauthor=true&cauthor_uid=21320734), [Park JT](http://www.ncbi.nlm.nih.gov/pubmed?term=Park%20JT%5BAuthor%5D&cauthor=true&cauthor_uid=21320734), [Kim JK](http://www.ncbi.nlm.nih.gov/pubmed?term=Kim%20JK%5BAuthor%5D&cauthor=true&cauthor_uid=21320734), [Kim SJ](http://www.ncbi.nlm.nih.gov/pubmed?term=Kim%20SJ%5BAuthor%5D&cauthor=true&cauthor_uid=21320734), [Oh HJ](http://www.ncbi.nlm.nih.gov/pubmed?term=Oh%20HJ%5BAuthor%5D&cauthor=true&cauthor_uid=21320734), [Yoo DE](http://www.ncbi.nlm.nih.gov/pubmed?term=Yoo%20DE%5BAuthor%5D&cauthor=true&cauthor_uid=21320734), [Han SH](http://www.ncbi.nlm.nih.gov/pubmed?term=Han%20SH%5BAuthor%5D&cauthor=true&cauthor_uid=21320734), [Yoo TH](http://www.ncbi.nlm.nih.gov/pubmed?term=Yoo%20TH%5BAuthor%5D&cauthor=true&cauthor_uid=21320734), [Kang SW](http://www.ncbi.nlm.nih.gov/pubmed?term=Kang%20SW%5BAuthor%5D&cauthor=true&cauthor_uid=21320734). (2011) Renal outcomes in patients with type 2 diabetes with or without coexisting non-diabetic renal disease. Diabetes Res Clin Pract. 92:198-204.
6. [Chia YC](http://www.ncbi.nlm.nih.gov/pubmed?term=Chia%20YC%5BAuthor%5D&cauthor=true&cauthor_uid=23259489), [Ching SM](http://www.ncbi.nlm.nih.gov/pubmed?term=Ching%20SM%5BAuthor%5D&cauthor=true&cauthor_uid=23259489). (2012) Hypertension and the development of New onset chronic kidney disease over a 10 year period: a retrospective cohort study in a primary care setting in Malaysia, BMC Nephrology. 13:173
7. Hanratty R, Chonchol M, Havranek EP, Powers JD, Dickinson LM, Ho PM, Magid DJ, and Steiner JF (2011) Relationship between Blood Pressure and Incident Chronic Kidney Disease in Hypertensive Patients. Clin J Am Soc Nephrol 6: 2605-11.
8. [Kanno A](http://www.ncbi.nlm.nih.gov/pubmed?term=Kanno%20A%5BAuthor%5D&cauthor=true&cauthor_uid=22510379), [Kikuya M](http://www.ncbi.nlm.nih.gov/pubmed?term=Kikuya%20M%5BAuthor%5D&cauthor=true&cauthor_uid=22510379), [Ohkubo T](http://www.ncbi.nlm.nih.gov/pubmed?term=Ohkubo%20T%5BAuthor%5D&cauthor=true&cauthor_uid=22510379), [Hashimoto T](http://www.ncbi.nlm.nih.gov/pubmed?term=Hashimoto%20T%5BAuthor%5D&cauthor=true&cauthor_uid=22510379), [Satoh M](http://www.ncbi.nlm.nih.gov/pubmed?term=Satoh%20M%5BAuthor%5D&cauthor=true&cauthor_uid=22510379), [Hirose T](http://www.ncbi.nlm.nih.gov/pubmed?term=Hirose%20T%5BAuthor%5D&cauthor=true&cauthor_uid=22510379), [Obara T](http://www.ncbi.nlm.nih.gov/pubmed?term=Obara%20T%5BAuthor%5D&cauthor=true&cauthor_uid=22510379), [Metoki H](http://www.ncbi.nlm.nih.gov/pubmed?term=Metoki%20H%5BAuthor%5D&cauthor=true&cauthor_uid=22510379), [Inoue R](http://www.ncbi.nlm.nih.gov/pubmed?term=Inoue%20R%5BAuthor%5D&cauthor=true&cauthor_uid=22510379), [Asayama K](http://www.ncbi.nlm.nih.gov/pubmed?term=Asayama%20K%5BAuthor%5D&cauthor=true&cauthor_uid=22510379), [Shishido Y](http://www.ncbi.nlm.nih.gov/pubmed?term=Shishido%20Y%5BAuthor%5D&cauthor=true&cauthor_uid=22510379), [Hoshi H](http://www.ncbi.nlm.nih.gov/pubmed?term=Hoshi%20H%5BAuthor%5D&cauthor=true&cauthor_uid=22510379), [Nakayama M](http://www.ncbi.nlm.nih.gov/pubmed?term=Nakayama%20M%5BAuthor%5D&cauthor=true&cauthor_uid=22510379), [Totsune K](http://www.ncbi.nlm.nih.gov/pubmed?term=Totsune%20K%5BAuthor%5D&cauthor=true&cauthor_uid=22510379), [Satoh H](http://www.ncbi.nlm.nih.gov/pubmed?term=Satoh%20H%5BAuthor%5D&cauthor=true&cauthor_uid=22510379), [Sato H](http://www.ncbi.nlm.nih.gov/pubmed?term=Sato%20H%5BAuthor%5D&cauthor=true&cauthor_uid=22510379), [Imai Y](http://www.ncbi.nlm.nih.gov/pubmed?term=Imai%20Y%5BAuthor%5D&cauthor=true&cauthor_uid=22510379). (2012) Pre-hypertension as a significant predictor of chronic kidney disease in a general population: the Ohasama Study. Nephrol Dial Transplant. 27: 3218-3223
9. Segura JN, Campo C, Gil P, Rolda CN,Vigil L, Rodicio JL, And Ruilope LM. (2004) Development Of Chronic Kidney Disease and Cardiovascular Prognosis in Essential Hypertensive Patients. J Am Soc Nephrol 15: 1616-1622.
10. Hanratty R, Chonchol M, Dickinson LM, Beaty BL, Estacio RO, MacKenzie TD, Hurley LP, Linas SL, Steiner JF and Havranek EP. (2010), Incident chronic kidney disease and the rate of kidney function decline in individuals with hypertension. Nephrol Dial Transplant 25: 801-807.
11. Schlessinger SD, Tankersley MR, Curtis JJ (1994). Clinical documentation of end stage renal disease due to hypertension. Am J Kidney Dis. 23:655–60.
12. Dhaun N, Bellamy CO, Cattran DC and Kluth DC. (2014) Utility of renal biopsy in the clinical management of renal disease. Kidney Int. 85:1039-48.
13. Rivera F, López-Gómez JM, Pérez-García R. (2004). Spanish Registry of Glomerulonephritis, Clinico-pathologic correlations of renal pathology in Spain. Kidney Int*.*  66:898-904.
14. Pei Y, Obaji J, Dupuis A, Paterson AD, Magistroni R, Dicks E, Parfrey P, Cramer B, Coto E, Torra R, San Millan JL, Gibson R, Breuning M, Peters D, Ravine D (2009). Unified criteria for ultrasonographic diagnosis of ADPKD. J Am Soc Nephrol. 20:205-12.
15. Taylor M, Johnson AM, Tison M, Fain P, Schrier RW. (2005) Earlier diagnosis of autosomal dominant polycystic kidney disease: importance of family history and implications for cardiovascular and renal complications. Am J Kidney Dis. 46:415-23.
