# Supplementary material for: Independent Role of Underlying Kidney Disease on Renal Prognosis of Patients with Chronic Kidney Disease under Nephrology Care
Source: PLoS One. 2015 May 20;10(5):e0127071. doi: 10.1371/journal.pone.0127071 (PMC4439030; doi:10.1371/journal.pone.0127071)
Supplement: S1 Table — (DOCX) [file pone.0127071.s002.docx]

TABLE**.**  Multivariable Cox models of determinants of the combined renal endpoint using different cut-off values for defining control of hypertension, anemia and proteinuria

|  | **Unadjusted** | |  | **Model 1** | |  | **Model 2** | | |
| --- | --- | --- | --- | --- | --- | --- | --- | --- | --- |
|  | HR | 95% CI |  | HR | 95% CI |  | HR | 95% CI |  |
| Cause of kidney disease |  |  |  |  |  |  |  |  |  |
| HTN | Ref. |  |  | Ref. |  |  | Ref. |  |  |
| DN | 2.72 | 1.84-4.03 |  | 2.44 | 1.63-3.66 |  | 1.89 | 1.23-2.90 |  |
| GN | 1.54 | 1.00-2.36 |  | 2.38 | 1.46-3.87 |  | 1.58 | 0.94-2.66 |  |
| TIN | 1.33 | 0.75-2.34 |  | 1.61 | 0.86-3.01 |  | 1.49 | 0.80-2.80 |  |
| PKD | 3.64 | 2.03-6.53 |  | 5.01 | 2.57-9.74 |  | 5.85 | 2.97-11.5 |  |
| Blood pressure goal |  |  |  |  |  |  |  |  |  |
| *Unachieved at month 12* | Ref. |  |  | Ref. |  |  | Ref. |  |  |
| *Achieved only at month 12* | 0.54 | 0.37-0.79 |  | 0.47 | 0.32-0.71 |  | 0.48 | 0.32-0.73 |  |
| *Achieved at both visits* | 0.41 | 0.27-0.62 |  | 0.42 | 0.27-0.65 |  | 0.46 | 0.29-0.71 |  |
| Hemoglobin goal |  |  |  |  |  |  |  |  |  |
| *Unachieved at month 12* | Ref. |  |  | Ref. |  |  | Ref. |  |  |
| *Achieved only at month 12* | 0.53 | 0.30-0.92 |  | 0.45 | 0.26-0.80 |  | 0.59 | 0.33-1.06 |  |
| *Achieved at both visits* | 0.29 | 0.20-0.41 |  | 0.38 | 0.26-0.56 |  | 0.42 | 0.29-0.63 |  |
| Proteinuria goal |  |  |  |  |  |  |  |  |  |
| *Unachieved at month 12* | Ref. |  |  | Ref. |  |  | Ref. |  |  |
| *Achieved only at month 12* | 0.56 | 0.34-0.93 |  | 0.57 | 0.35-0.95 |  | 0.59 | 0.35-0.99 |  |
| *Achieved at both visits* | 0.34 | 0.24-0.48 |  | 0.36 | 0.25-0.54 |  | 0.41 | 0.27-0.63 |  |

Model 1: diagnoses and goals are separately adjusted for main covariates (age, gender, history of CV disease, BMI, eGFR, use of RAS inhibitors). Model 2: fully adjusted (adjusted for main covariates of model 1, diagnoses and therapeutic goals). **Therapeutic goals: Proteinuria <1 g/24h; Blood pressure <140/90 mmHg; Hemoglobin 10-12 g/dL during ESA therapy or Hb spontaneously ≥11 g/dL.** HTN, hypertensive nephropathy; DN, diabetic nephropathy; GN, glomerulonephritis; PKD, autosomal polycystic kidney disease; TIN, tubulointerstitial nephropathy
